# Exploration of Alternative Splicing Events in Mesenchymal Stem Cells from Human Induced Pluripotent Stem Cells

**DOI:** 10.3390/genes12050737

**Published:** 2021-05-13

**Authors:** Ji-Eun Jeong, Binna Seol, Han-Seop Kim, Jae-Yun Kim, Yee-Sook Cho

**Affiliations:** 1 Stem Cell Research Laboratory, Immunotherapy Research Center, Korea Research Institute of Bioscience and Biotechnology, 125 Gwahak-ro, Yuseong-gu, Daejeon 34141, Korea; happy_jje@naver.com (J.-E.J.); nana8610@kribb.re.kr (B.S.); khs1109@kribb.re.kr (H.-S.K.); jameskim@kribb.re.kr (J.-Y.K.); 2 Department of Bioscience, KRIBB School, University of Science & Technology, 113 Gwahak-ro, Yuseong-gu, Daejeon 34113, Korea

**Keywords:** human induced pluripotent stem cells, differentiation, mesenchymal stem cells, RNA sequencing, transcriptome, alternative splicing

## Abstract

Although comparative genome-wide transcriptomic analysis has provided insight into the biology of human induced pluripotent stem cell-derived mesenchymal stem cells (iMSCs), the distinct alternative splicing (AS) signatures of iMSCs remain elusive. Here, we performed Illumina RNA sequencing analysis to characterize AS events in iMSCs compared with tissue-derived MSCs. A total of 4586 differentially expressed genes (|FC| > 2) were identified between iMSCs and umbilical cord blood-derived MSCs (UCB-MSCs), including 2169 upregulated and 2417 downregulated genes. Of these, 164 differentially spliced events (BF > 20) in 112 genes were identified between iMSCs and UCB-MSCs. The predominant type of AS found in iMSCs was skipped exons (43.3%), followed by retained introns (19.5%), alternative 3′ (15.2%) and 5′ (12.8%) splice sites, and mutually exclusive exons (9.1%). Functional enrichment analysis showed that the differentially spliced genes (|FC| > 2 and BF > 20) were mainly enriched in functions associated with focal adhesion, extracellular exosomes, extracellular matrix organization, cell adhesion, and actin binding. Splice isoforms of selected genes including *TRPT1*, *CNN2*, and *AP1G2*, identified in sashimi plots, were further validated by RT-PCR analysis. This study provides valuable insight into the biology of iMSCs and the translation of mechanistic understanding of iMSCs into therapeutic applications.

## 1. Introduction

Human tissue-derived multipotent mesenchymal stem cells (MSCs) have emerged as one of the most promising cell types for the treatment of a broad spectrum of diseases, owing to their ease of isolation and growth in culture and tissue regeneration, and immune tolerance abilities [1,2]. Despite research efforts, cell-to-cell variation in the quality and phenotype of MSCs depending on the cell source and preparation protocol remains an obstacle to their widespread therapeutic application. Recently, human induced pluripotent stem cells (hiPSCs) have received attention as an attractive cell source for large scale production of homogeneous MSCs for cell therapy and tissue-engineering applications, owing to their unlimited self-renewal capability and strong ability to differentiate into MSCs, and the absence of ethical concerns surrounding their use [1,3]. 

At the cellular and functional levels, MSCs derived from hiPSCs (iMSCs) acquire properties similar to those of human tissue-derived MSCs (tMSCs), including multi-lineage differentiation capability, immunomodulatory and immune suppressive activity, and therapeutic efficacy [1,2,4,5]. Comparably to tMSCs, exogenous iMSCs have regenerative therapeutic potential for various diseases including ischemia [6], bone defects [7], inflammatory bowel disease [8], chronic obstructive pulmonary disease [9], mitochondrial damage-induced retinal ganglion cell degeneration [10], and acute kidney injury [11]. Beyond direct iMSC-mediated effects, iMSCs have been demonstrated to have therapeutic paracrine effects, thus resulting in an attenuation of cardiomyopathy [12] and rejuvenation in aging [13]. In another application, disease-specific iMSCs have provided unparalleled opportunities as human cell-based disease models for pathological research, drug screening, and toxicity testing to discover novel therapeutic strategies for treating a variety of mesenchymal diseases, such as osteogenesis imperfecta [14], Hutchinson–Gilford progeria syndrome [15], axial spondyloarthritis [16], and fibrous dysplasia [17].

At the molecular level, genome-wide transcriptomic comparison through RNA sequencing (RNA-Seq) analysis of various isogenic pairs, including fetal femur-derived MSCs, bone marrow-derived MSCs [13], and Wharton’s jelly-derived MSCs [18], together with the iMSCs derived from them, has revealed that iMSCs largely recapitulate the global transcriptomic signatures of tMSCs. Comparative transcriptomic analysis has also indicated some differences in transcriptomic profiles between tMSCs and iMSCs [4,13,18,19,20]. iMSCs, as compared with tMSCs, display a distinct transcriptional profile associated with rejuvenation signatures at the genetic and epigenetic levels, independently of donor age and cell source [13,21]. To define the iMSC gene repertoire associated with this cell type’s distinct characteristics and function, understanding the iMSC-specific gene expression controlled at both the transcriptional and post-transcriptional levels is important. However, most studies of iMSCs have focused on the analysis of gene expression at the transcriptional level, whereas gene expression at the post-transcriptional level is much less clear. Post-transcriptional alternative splicing (AS) of precursor mRNAs, a major gene regulatory mechanism contributing to RNA and protein diversity, modulates almost all fundamental biological processes including tissue development and identity [22,23,24], as well as stem cell identity and stemness [25,26,27]. The regulatory roles of AS in MSCs that govern MSC identity, differentiation, and aging have been demonstrated [28,29,30,31,32]. However, current understanding of global AS events and AS-derived mRNA isoforms in transcriptomes of iMSCs remains largely lacking. 

In this study, we comprehensively profiled the iMSC transcriptome through RNA-Seq analysis to investigate the AS event profiles of iMSCs compared with umbilical cord blood-derived mesenchymal stem cells (UCB-MSCs). Understanding AS events of iMSCs should help decipher the gene regulatory network controlling iMSC fate and the diverse biological functions underlying the potential therapeutic potential of iMSCs.

## 2. Materials and Methods

### 2.1. Cell Culture

CRL-2097 human normal skin fibroblasts (HFs) were purchased from the American Type Culture Collection (ATCC: Manassas, VA, USA). Human UCB-MSCs were purchased from the Japanese Collection of Research Bioresources cell bank (JCRB1109, Osaka, Japan) and ATCC (PCS-500-010, Manassas, VA, USA). The HFs were cultured in α-MEM (GIBCO, Carlsbad, CA, USA) fibroblast medium containing 10% fetal bovine serum, GIBCO), 1% minimum essential medium non-essential amino acid (GIBCO), 1% penicillin/streptomycin (GIBCO), and 1% sodium pyruvate (GIBCO). UCB-MSCs were cultured in MesenCult™-ACF Medium (STEMCELL technologies, Cambridge, MA, USA). The hiPSCs reprogrammed from HFs by using Sendai-virus vectors encoding Oct4, Sox2, Klf4, and c-Myc [33], or episomal vectors (Episomal iPSC reprogramming vectors, Thermo Fisher Scientific, Waltham, MA, USA) [34], were cultured with mTeSR1 (STEMCELL Technologies) medium. 

### 2.2. Differentiation of hiPSCs into iMSCs

The hiPSCs were differentiated into MSCs with a STEMdiff^TM^ Mesenchymal Progenitor kit (STEMCELL Technologies) for 3 weeks, according to the manufacturer’s instructions.

### 2.3. Characterization of MSCs

One million iMSCs and UCB-MSCs were stained with PE-conjugated CD73, PE-conjugated CD105, PE-conjugated CD90 (Miltenyi Biotec, Somerville, MA, USA), PE-conjugated CD34, and FITC-conjugated CD45 (BioLegend, San Diego, CA, USA) for 30 min at 4 °C. Stained cells were washed twice in PBS, and FACS was performed with a BD Accuri C6 flow cytometer (BD Biosciences, San Diego, CA, USA). The antibodies used are listed in Supplementary Table S6.

### 2.4. RNA Isolation and Reverse Transcription-Polymerase Chain Reaction (RT-PCR) 

Total RNA was extracted from iMSCs and UCB-MSCs with an RNeasy Mini Kit (Qiagen, Valencia, CA, USA) according to the manufacturer’s instructions. The quality of total RNA was verified with an Agilent 2100 bioanalyzer and an RNA 6000 Nano Chip (Agilent Technologies, Amstelveen, The Netherlands). RNA quantification was accomplished with an ND-2000 spectrophotometer (Thermo Inc., Waltham, MA USA). For RT-PCR analysis, complementary DNA (cDNA) was transcribed from total RNA with a SuperScript^®^ VILO cDNA Synthesis Kit and Master Mix (Thermo Fisher Scientific). RT-PCR was performed with 5× Taq-PCR Mix (GenoTech Corp., Daejeon, Korea) to evaluate the AS events in the MSCs, including those of TRNA phosphotransferase 1 (*TRPT1*), calponin 2 (*CNN2*), and AP-1 complex subunit gamma-like 2 (*AP1G2*). Expression of the housekeeping gene human glyceraldehyde-3-phosphate dehydrogenase (*GAPDH*) was used as a control. PCR product bands stained with ethidium bromide were visualized under ultraviolet light. The RT-PCR results were quantified in ImageJ software (NIH, Bethesda, MD, USA). The primer list is shown in Supplementary Table S7.

### 2.5. Library Construction and Sequencing

Libraries were constructed from 2 μg of total RNA with a SMARTer Stranded RNA-Seq Kit (Clontech Laboratories, Inc., Mountain View, CA, USA), according to the manufacturer’s instructions. The mRNA was isolated with a Poly(A) RNA Selection Kit (LEXOGEN, Inc., Vienna, Austria) and used for cDNA synthesis and shearing. Indexing was performed with Illumina indexes 1–12. The enrichment step was performed by PCR. Subsequently, libraries were verified with an Agilent 2100 bioanalyzer (DNA High Sensitivity Kit) to evaluate the mean sizes of the fragments. Quantification was performed with a library quantification kit and a StepOne Real-Time PCR System (Thermo Fisher Scientific). The HiSeq 2500 Sequencing System (Illumina, Inc., San Diego, CA, USA) was used for paired-end 100 sequencing. High-throughput sequencing and sequence analysis were conducted by ebiogen Inc. (Seoul, Korea). The raw reads were deposited in NCBI’s Sequence Read Archive under accession numbers SRR13907520 (iMSC) and SRR13907525 (UCB-MSC), respectively.

### 2.6. Data Analysis

Reads obtained from RNA-Seq were aligned to the human reference genome in TopHat software v.2.1.0 [35]. The read count data obtained with Bedtools [36] were processed with the quantile normalization method by using EdgeR v.3.20.1 in R (R development Core Team, 2016) and Bioconductor [37]. The read counts were converted to log2 counts per million, and a|log2-fold change (FC)| ≥ 2 at *p* value < 0.05 was considered as the cutoff value for differentially expressed gene (DEG) selection. The alignment files were processed with Cufflinks (v.2.2.1) to determine transcript abundance and identify differentially expressed, spliced, or transcriptionally regulated genes. The expression output values from Cufflinks were normalized with EdgeR, and mixture of isoforms software (MISO) were used to perform the splicing analysis. The log2(FC) of the normalized reads per kilobase per million (RPKM) was calculated separately for each library with DEGs or differentially spliced genes (DSGs). The expression values of libraries were normalized with the upper quartile normalization method in the EdgeR package before clustering of gene and isoform profiles. Gene expression FC values were analyzed with ExDEGA (Excel-based DEG Analysis tool, ebiogen Inc.). Gene Ontology (GO) function and Kyoto Encyclopedia of Genes and Genomes (KEGG) pathway enrichment analyses of DEGs were performed with the gene set enrichment analysis (GSEA, http://software.broadinstitute.org/gsea/index.jsp, accessed on 19 September 2020) [38,39] and DAVID (http://david.abcc.ncifcrf.gov/, accessed on 18 September 2020) online databases [40,41]. GO functional classification of DEGs was defined on the basis of the QuickGo database (https://www.ebi.ac.uk/QuickGO/, accessed on 10 August 2020). We used DAVID v.6.8 and KEGG v.4.0 in this study. Reactome analysis (https://reactome.org/, accessed on 19 September 2020) was performed for pathway enrichment analysis. 

### 2.7. Analysis of Alternative Splicing Events with the MISO Package

The MISO package v.0.5.4 with the default options was used to detect differentially spliced exons between samples. Read alignment files (BAM) produced by TopHat and GFF3 annotation of human genome (hg19) alternative events v1.0, according to the MISO reference manual [42], were used for MISO analysis. Transcript abundance values estimated with Cufflinks for AS events were classified into five types: skipped exons (SEs), alternative 3′ and 5′ splice sites (A3SS and A5SS, respectively), mutually exclusive exons (MXEs), and retained introns (RIs). Sashimi plots from MISO were used to visualize the alternatively spliced exon analysis results [33]. When comparing iMSCs and UCB-MSCs, we used two values to identify differentially spliced events: (1) difference in percent spliced in (Ψ) values, with the cutoff |Δψ| > 0.1, representing the inclusion ratio of an isoform in a library, and (2) Bayes factor (BF; cutoff > 20), as a measure of the confidence of an event being differentially spliced between iMSCs and UCB-MSCs. For individual splicing patterns of selected genes, the sashimi plots were plotted from the bam files by using the sashimi plot function in MISO. 

## 3. Results

### 3.1. Identification of DEGs between hiMSCs and UCB-MSCs

**Figure 1:**
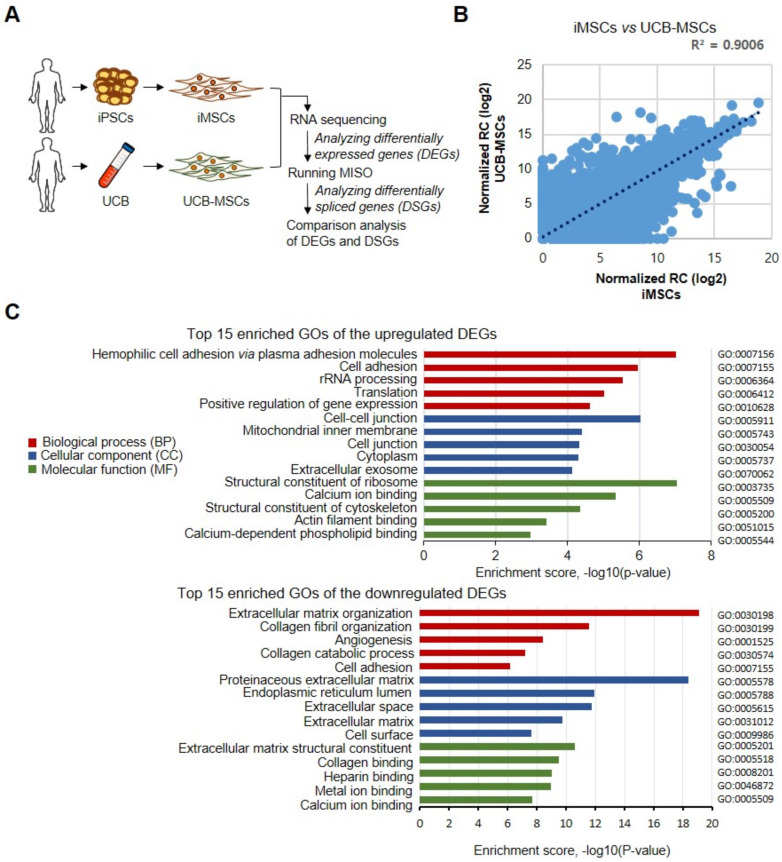
Comparative analysis of DEGs between iMSCs and UCB-MSCs. (**A**) A schematic view of the experimental design of comparative transcriptomic analysis by RNA-Seq for comparison of iMSCs versus UCB-MSCs. (**B**) Scatter plots show correlations between iMSCs and UCB-MSCs. R2 indicates the coefficient of determination. (**C**) The top 15 GO terms of up- regulated and down-regulated DEGs. GO analysis classified the DEGs into three groups: red, biological process; blue, cellular component; and green, molecular function. The x-axis indicates the enrichment scores [-log10 (*p* value), *p*-value cut-off of 0.05] for each GO term. The y-axis represents the detailed classification of the GO or KEGG pathway terms.

To gain additional insights into hiMSCs, we performed transcriptomic analysis by RNA-Seq on hiMSCs derived from integration-free hiPSCs reprogrammed by Sendai virus vectors carrying sequences of human Oct4, Sox2, Klf4, and c-Myc from skin fibroblasts. The identity of MSCs used in this study was confirmed by FACS analysis showing that the cells were positive for the MSC-associated surface makers CD73, CD105, and CD90 and negative for the hematopoietic makers CD34 and CD45 (Supplementary Figure S1). Whole transcriptomic profiles of hiMSCs were compared with the transcriptomes of UCB-MSCs (Figure 1A), and DEGs were identified by application of the screening thresholds of 2-fold changes (FC) with *p* value < 0.05 (Figure 1B). Comparison of the RNA-Seq results revealed a strong positive correlation between DEGs of hiMSCs and UCB-MSCs (R^2^ = 0.9; Figure 1B). Among all 4586 DEGs (24.5% of total genes), 2169 (11.6% of total genes) were upregulated, and 2417 (12.9% of total genes) were downregulated in iMSCs compared with UCB-MSCs. The 30 most significantly upregulated and downregulated DEGs are shown in Supplementary Tables S1 and S2, respectively. The upregulated genes included *PAX8*, *UBE2Q2P2*, *L1CAM*, *KRT19*, *NDN*, *FOXL2*, *CLDN1*, *GOLGA6L10*, *SYNDIG1*, *SPP1*, *DSC3*, *PDGFB*, *IGFN1*, *CPXM1*, and *GALNT14* (Supplementary Table S1). The downregulated genes included *POSTN*, *SULF1*, *ADAMTS2*, *FAP*, *BGN*, *OLFM2*, *COL1A2*, *GALNT5*, *COL5A3*, *ELN*, *SIX2*, *NUPR1*, *LMO2*, *NPR3*, and *HEY2* (Supplementary Table S2). 

### 3.2. Functional Analysis of DEGs 

The biological importance of 4586 DEGs was further explored with GO term enrichment and KEGG pathway analysis with DAVID, with *p* < 0.05 as the threshold. Upregulated DEGs were enriched in 236 GO terms and classified into categories of BP with 143 GO terms, CC with 55 GO terms, and MF with 38 GO terms. The downregulated DEGs were enriched in 245 terms in the category BP, 51 terms in the category CC, and 77 terms in the category MF. The top five GO terms of BP, CC, and MF of the upregulated and downregulated DEGs are shown in Figure 1C. The upregulated DEGs were particularly enriched in the terms “hemophilic cell adhesion via plasma membrane adhesion molecules (BP, GO:0007156),” “cell adhesion (BP, GO:0007155),” “rRNA processing (BP, GO:0006364),” “cell-cell junction (CC, GO:0005911),” “mitochondrial inner membrane (CC, GO:0005743),” “cell junction (CC, GO:0030054),” “structural constituent of ribosome (MF, GO:0003735),” “calcium ion binding (MF, GO:0005509),” and “structural constituent of cytoskeleton (MF, GO:0005200)” (Figure 1C). The downregulated DEGs were significantly enriched in “extracellular matrix organization (BP, GO:0030198),” “collagen fibril organization (BP, GO:0030199),” “angiogenesis (BP, GO:0001525),” “proteinaceous extracellular matrix (CC, GO:0005578),” “endoplasmic reticulum lumen (CC, GO:0005788),” “extracellular space (CC, GO:0005615),” “extracellular matrix structural constituent (MF, GO:0005201),” “collagen binding (MF, GO:0005518),” and “heparin binding (MF, GO:0008201)” (Figure 1C). On the basis of KEGG pathway analysis, the top five enriched pathways of upregulated DEGs were associated with “ribosome,” “pathogenic Escherichia coli infection,” “cell adhesion molecules (CAMs),” “Rap1 signaling pathway,” and Alzheimer’s disease” (Supplementary Figure S1). The top five most significantly enriched pathways in downregulated DEGs were “ECM-receptor interaction,” focal adhesion,” “protein digestion and absorption,” “PI3K-Akt signaling pathway,” and “proteoglycans in cancer” (Supplementary Figure S1). 

### 3.3. Identification of DSEs between hiMSCs and UCB-MSCs

**Figure 2:**
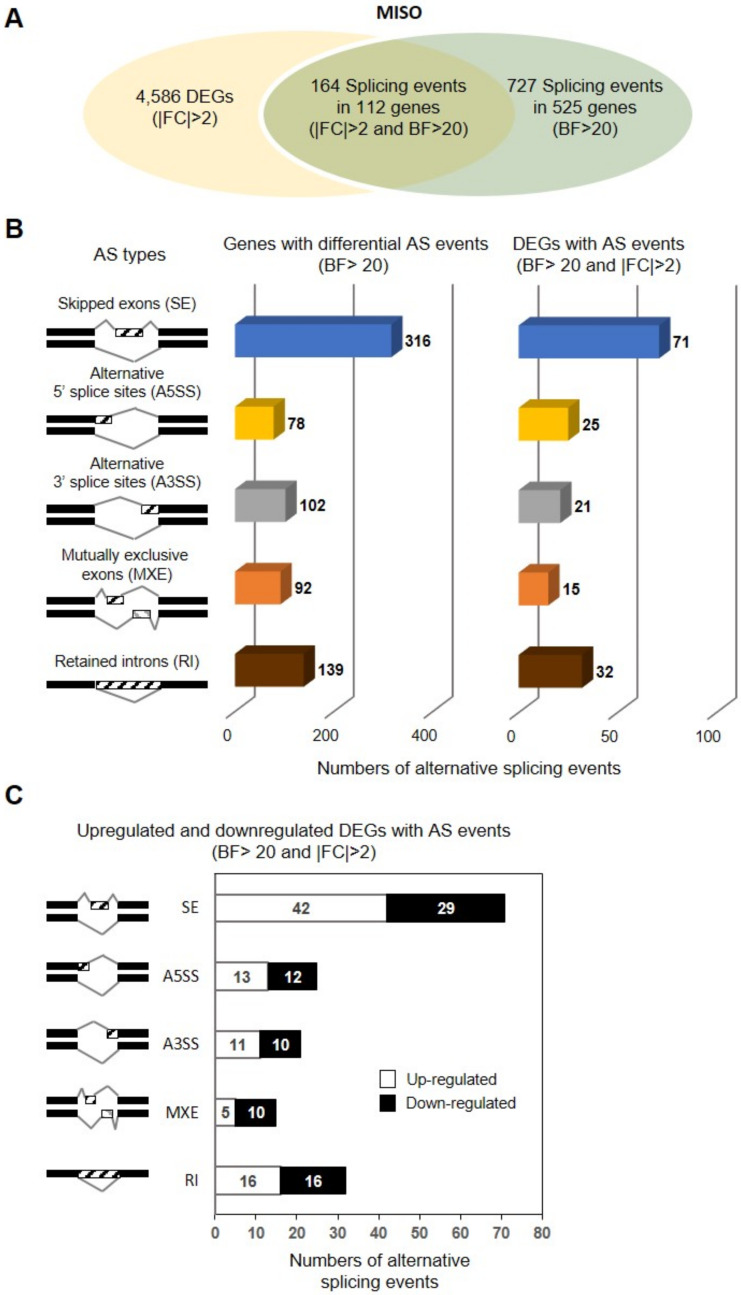
Comparative analysis of DSEs between iMSCs and UCB-MSCs. (**A**) Venn diagram showing the overlap of DEGs and DSEs detected by RNA-Seq in a comparison of iMSCs versus UCB-MSCs. AS events with BF > 20 were used for further analysis. (**B**) The number of DEGs with indicated AS events. (**C**) The number of upregulated and downregulated DEGs with indicated AS events. The x-axis represents the number of transcripts for each type of AS events. The y-axis represents five types of AS events, including SE, MXE, A5SS, A3SS, and RI.

To understand the transcriptomic diversity and gene expression dynamics in hiMSCs, we further compared AS events between hiMSCs and UCB-MSCs by using the exon-centric MISO method (v. 0.4.1 with default parameters). Significant differentially spliced events (DSEs) were determined with BF of at least 20. A total of 727 DSEs, including all five types of AS events (316 SEs, 102 A3SS, 78 A5SS, 92 MXEs, and 139 RIs), were identified from 525 genes (Figure 2A,B). Of all 525 genes with AS events, 22.3% of the DSGs, corresponding to 112 genes, overlapped with DEGs (|log2(FC)| ≥ 2 and *p* < 0.05) (Figure 2A,B), thus suggesting that those genes are potentially regulated by both transcription and AS. Of the AS events identified, SE events were the most predominant types, constituting 42.8% of all 71 AS events (42 up-regulated and down-regulated), followed by RIs (19.3%; 16 up-regulated and 16 down-regulated), A5SS (15.1%; 13 up-regulated and 12 down-regulated), A3SS (12.7%; 11 up-regulated and 10 down-regulated), and MXEs (9.0%; 5 up-regulated and 10 down-regulated) (Figure 2B,C). Lists of up-regulated and down-regulated DEGs with each AS event are provided in Supplementary Table S3. 

### 3.4. Functional Analysis of DSGs 

**Figure 3:**
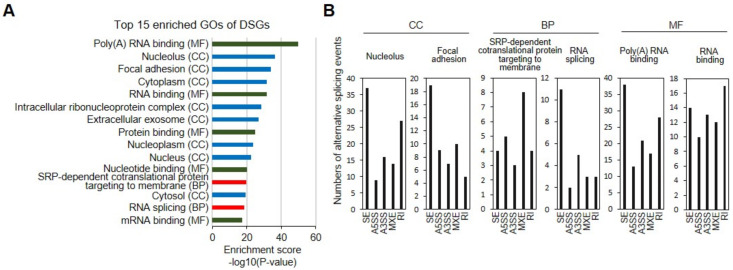
Comparative GO analysis of genes with AS events between iMSCs and UCB-MSCs. (**A**) Top 15 GO terms of genes with AS events. The bar chart showing 15 GO terms significantly enriched in iMSCs. The x-axis indicates the enrichment scores [−log10 (*p* value), *p*-value cut-off of 0.05] for each GO term. The y-axis represents each detailed classification of GO terms. (**B**) The numbers of the five types of AS events for each indicated GO term.

Next, functional GO, KEGG, and/or Reactome enrichment analyses were performed on DSGs (BF > 20) and DEGs with AS events (|log2(FC)| ≥ 2, *p* < 0.05, and BF > 20) by using GSEA and DAVID (Figure 3). We determined through DAVID analysis that DSGs (BF > 20) were enriched in the GO pathways “nucleolus (CC),” “focal adhesion (CC),” and “SRP-dependent cotranslational protein targeting to membrane (BP),” “RNA splicing (BP),” “poly (A) RNA binding (MF),” “RNA binding (MF),” “cytoplasm (CC),” “mRNA metabolic process,” “ribonucleoprotein complex,” and “cell substrate junction” (Figure 3A). All five types of AS events were observed in GO annotated DSGs (Figure 3B). For the most enriched CC GO term “nucleus,” SE events (37) were the most abundant, followed by RIs (27), A3SS (16), MXEs (14), and A3SS (9) (Figure 3B). Similarly, SEs (38) featured the greatest number of AS events in the MF GO term “poly (A) RNA binding,” followed by RI (28), A3SS (27), MXE (17), and A3SS (13) (Figure 3B). For the BP GO term “SRP-dependent cotranslational protein targeting to membrane,” MXE (8) was most prevalent, followed by A5SS (5), SE, RI (4), and A3SS (3) (Figure 3B). Lists of GO annotated DSGs with each AS type are provided in Supplementary Table S4. We also found that DSGs were enriched in the KEGG pathways “spliceosome,” “ribosome,” “lysosome,” “focal adhesion,” and “tight junction,” and in Reactome pathways “metabolism of RNA,” “nervous system development,” post-translational protein modification,” “translation,” and “SRP-dependent cotranslational protein targeting to membrane” (Supplementary Table S5). 

**Figure 4:**
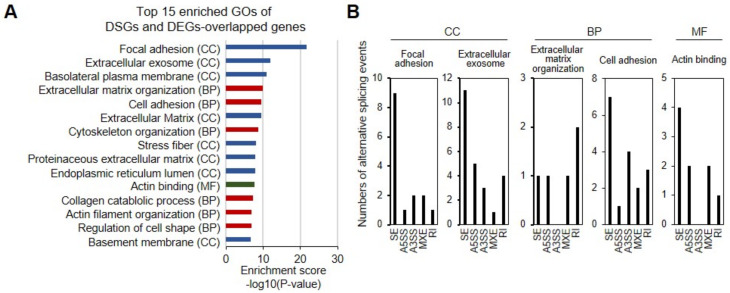
Comparative GO analysis of DEGs with AS events between iMSCs and UCB-MSCs. (**A**) The top 15 GO terms for DEGs with AS events. The bar chart shows 15 GO terms significantly enriched in iMSCs. The x-axis indicates the enrichment scores [-log10 (*p* value), *p*-value cut-off of 0.05] for each GO term. The y-axis shows the detailed classification of each GO term. (**B**) The number of the five types of AS events for each indicated GO term.

Further GO analysis identified the top 15 GO terms significantly enriched for DEGs with AS events, functionally involved in “focal adhesion (CC),” “extracellular exosome (CC),” “extracellular matrix organization (BP),” “cell adhesion (BP),” and “actin binding (MF),” on the basis of DAVID analysis (Figure 4A). The most enriched CC GO term, “focal adhesion,” included genes with SEs (*ACTB*, *BCAR1*, *CD46*, *CNN2*, *FAT1*, *FHL1*, *NRP1*, and *USP33*), A3SS (*FBLIM1* and *PDLIM7*), MXEs (*ITGB5* and *RPS3*), A5SS (*DAG1*), or RIs (*TNS3*) (Figure 4B). The other enriched CC GO term, “extracellular exosome (CC)”, contained genes with SEs (*ACTB*, *CNN2*, *EXTL2*, *FAM171A1*, *FAT1*, *LAMP2*, *PIN4*, and *TUBA1B*), A5SS (*CTSL*), A3SS (*COL12A1*, *PRNP*, and *SNRPB*), MXEs (*ITGB5*) or RIs (*BEND7*, *PKD2*, and *TGFBI*) (Figure 4B). For the most enriched BP GO term, “cell adhesion,” genes with SEs (*BCAR1*, *CD164*, *FAT1*, *KIAA1462*, and *SUSD5*), A5SS (*COL5A1*), A3SS (*CD164*, *COL12A1*, *COL5A1*, and *COL6A2*), MXEs (*COL5A1* and *ITGB5*), or RIs (*CD164* and *TGFBI*) were identified (Figure 4B). In addition, MF GO term, “actin binding,” included genes with SEs (*CNN2*, *DBN1*, and *TAGLN*), A5SS (*DAG1* and *TAGLN*), MXE (*TPM2* and *WASF3*), or RIs (*PDLIM5*) (Figure 4B). We expect that further functional investigation of candidate DSGs will contribute to a more in-depth understanding of MSC type-specific transcriptome signatures and regulation. 

### 3.5. Visualization and Validation of DSGs

**Figure 5:**
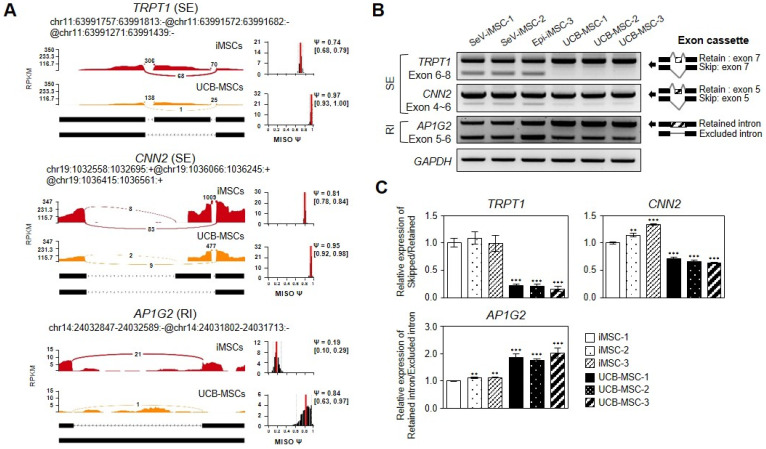
Sashimi plots and RT-PCR showing SE events in selected DSGs. (**A**) The sashimi plots of SE events in TRPT1 and CNN2, and RI events in AP1G2 were constructed with the MISO package. The vertical axis on the left side of the main panel shows the counts of RNA-Seq reads spanning the junctions in each region. The genomic coordinates for individual splice sites are shown at the top, and the schematic of each splicing event is shown at the bottom. Red indicates the iMSCs (C_MSC-1), and orange indicates the UCB-MSCs (C_MSC-3). The data are represented as reads per kilobase per million (RPKM). The estimated Ψ (red line) MISO values are shown in the right panels. (**B**) Representative AS events validated by RT-PCR analysis. RT-PCR was performed with primers in constitutive exons (dark gray in the scheme) of the three selected genes with SEs (TRPT1 and CNN2) and RIs (AP1G2). RT-PCR was performed with the same samples used in RNA-Seq. GAPDH was used as an internal control. (**C**) Quantitative analysis of the PCR results in B. The graphs show the ratio between isoforms with retained and skipped (or intron retention) exons. The data are relative to iMSC-1 and are shown as means ± SDs of three independent experiments. ** *p* < 0.01 and *** *p* < 0.001.

Using IGV sashimi plots, we visualized the differentially spliced exons along genomic regions of randomly selected representative genes with SEs (*TRPT1* and *CNN2*), RIs (*AP1G2*) (Figure 5A), A5SS (*TRPT1*), A3SS (*TRPT1*), or MXEs (*CALU*) (Supplementary Figure S3). Sashimi plots revealed that *TRPT1* featured multiple AS events, including SEs (Figure 5A), A5SS, and A3SS (Supplementary Figure S3). Subsequent semi-quantitative RT-PCR analysis confirmed the presence of multiple splice variants of selected genes in iMSCs (Figure 5B,C). The AS events that were validated by RT-PCR with the same samples (iMSC-1 and UCB-MSC-1) used in RNA-seq were consistent with our RNA-Seq results (Figure 5B). To further assess the quality and reproducibility of our data, we performed RT-PCR analysis in four additional independent MSC lines, including two iMSC lines and two UCB-MSCs. The two tested iMSC lines were produced from iPSCs derived through different reprogramming methods, on the basis of Sendai virus vectors and episomal vectors, from the same donor. The two UCB-MSCs were obtained from different donors and thus had different genetic backgrounds. Notably, similar PCR results were consistently obtained with six tested MSC lines for three selected genes (*TRPT1, CNN2,* and *AP1G2*), thus indicating that our AS event analysis results based on RNA-Seq data were reliable. Of note, according to the sashimi plot (Figure 5A), we cannot exclude the possibility that *CNN2* gene may give rise to another splice isoform in exon 5, potentially a minor splice isoform, and further validation is required.

## 4. Discussion

This work comprehensively characterized the differences in the gene expression profiles and splicing events of iMSCs and tMSCs through RNA-Seq and MISO analyses. Our global transcriptomic analysis of iMSCs compared with UCB-tMSCs revealed 4586 DEGs (24.5%), with annotated GO terms predominantly associated with cell structure and organization. Among the related DEGs, *COL1A1* (collagen Type I alpha 1 chain), *CTGF* (connective tissue growth factor), *FTH1* (ferritin heavy chain 1), *TIMP1* (tissue inhibitor matrix metalloproteinase 1), and *TGFBI* (transforming growth factor-beta induced) were significantly downregulated in iMSCs compared with UCB-MSCs, findings similar to those in other reports [20,43]. In agreement with our results, accumulating evidence suggests the presence of substantial differences between iMSCs and tMSCs in proliferation and differentiation capability and immunomodulatory potential [18,21,44], possibly as a result of different levels of molecular regulation at the transcriptome level, as revealed by genome-wide studies [13,18,21]. The numbers of DEGs among MSC lines appeared to reflect the different donors, sources, and preparation methods. Analysis of the DEGs between iMSCs and their isogenic UCB-MSCs revealed the variance of DEGs (2029–3065 genes), depending on the MSC differentiation methods and donors [18]. Bone marrow-derived MSCs from patients with aortic dissection and those from age-matched healthy donors have revealed 201 DEGs [45], whereas MSCs obtained from different tissues, including Wharton’s jelly and adipose tissue, have revealed 92 DEGs [46]. In addition, single-cell RNA-seq analysis has demonstrated the existence of subpopulations in MSCs, independently of donors and passages, by confirming the high variability of DEGs observed in MSC subsets [47].

In addition to identification of DEGs, investigation of transcriptome complexity and diversity on the basis of the tissue- or cell-specific expression of alternative transcripts has demonstrated that a global AS landscape of cellular genes reflects changes in stem cell identity and status [26,29,32]. Furthermore, a potential role of AS in MSCs has been suggested on the basis of the identification of MSC-specific, alternatively spliced isoforms of the SRRF (stromal RNA regulating factor) gene, encoding a novel member of the RRM subfamily of proteins [48], and the age-dependent, yet weak, splicing changes in aging MSCs compared with young MSCs [49]. In addition, one study has reported 53 hypoxia-dependent genes undergoing AS events, including upregulated *LEP*, *IL-11*, *IGFBP1*, *TEK*, *CA9*, *LOX4*, *HCK,* and *EGR2,* and downregulated *EFNA3*, *CORO7*, *FER1L5*, *MYH2,* and *ACAT1*, which are functionally associated with cell adhesion, migration, apoptosis, angiogenesis, and oxidation-reduction, thus describing the molecular effects of hypoxia on MSC biology and function [28]. However, sufficient understanding of AS events to fully define the gene expression regulation and transcriptome diversity responsible for fundamental and distinct iMSC characteristics remains lacking. Comparison of AS signatures and splicing differences between iMSCs and tMSCs have been neglected to date. 

Therefore, we performed AS profiling characterizing iMSCs and tMSCs as a paradigm for investigating iMSC identity and cell type-specific AS patterns. We identified a total of 727 DSEs corresponding to 525 genes when comparing iMSCs and UCB-tMSCs, thereby suggesting differences between iMSCs and UCB-tMSCs in AS patterns of expressed genes. We revealed the first profile of iMSC-specific AS events, classified into five distinct types: 316 (43.5%) SEs, 102 (14%) A3SS, 78 (10.7%) A5SS, 92 (12.7) MXEs, and 139 (19%) RIs (Figure 2B,C). Several genes, including TRPT1, featured more than one type of AS event in their sequences (Figure 5 and Supplementary Figure S2A and S2B). *TRPT1* (tRNA phosphotransferase) encodes the enzyme catalyzing the last step of tRNA splicing, thus indicating its potential role in modulation of iMSC-specific splicing regulation. GO analysis showed that these iMSC-specific isoforms are associated with a broad range of biological processes, including “nucleolus,” “focal adhesion,” “SRP-dependent cotranslational protein targeting to membrane,” “RNA splicing,” “poly (A) RNA binding,” “RNA binding,” “cytoplasm,” “mRNA metabolic process,” “ribonucleoprotein complex,” and “cell substrate junction,” thereby implying the complexity of transcriptional and post-transcriptional regulation of iMSCs. These results also indicate that divergence in AS patterns may substantially contribute to shaping iMSC-specific traits and may consequently result in distinct transcripts and protein isoforms. Notably, we determined that a total of 112 enriched DEGs (2.4% of all DEGs) in iMSCs underwent AS events. Of these, SE events (42.8%) were the major type of AS events, followed by RIs (19.3%), A5SS (15.1%), A3SS (12.7%), and MXEs (9.0%). The enriched GO terms indicated that the DEGs with AS events were enriched mainly in “focal adhesion (CC),” “extracellular exosome (CC),” “extracellular matrix organization (BP),” “cell adhesion (BP),” and “actin binding (MF)” (Supplementary Table S8). Semi-quantitative RT-PCR results performed in three independent sets of iMSCs and UCB-MSCs supported the integrity of our RNA-seq data to validate selected AS events. 

Further investigation focusing on donor-matched iMSCs and UCB-MSCs should enable more reliable/robust comparison among MSC types to clarify possible effects of inter-donor and inter-clonal variation. In addition, the future use of third-generation sequencing and long-read sequencing, which can overcome limitations in accuracy and throughput, is highly desirable for better experimental outcomes in cell or tissue type-specific analyses for sufficiently covering low-abundance transcripts, including minor splicing variants. 

## 5. Conclusions

Overall, our data provide the first reported profile of iMSC-specific transcriptome and splicing patterns, providing additional evidence of distinct gene repertoires characterizing iMSCs. Our study may contribute to providing a more stringent definition of iMSCs as an attractive alternative to tMSCs and a refined molecular portrait of iMSCs. Further research will be required to fully delineate the contributions of genes with iMSC-specific AS patterns on the biological functions and processes responsible for iMSC-specific traits. 

## Data Availability

Data is contained within the article or Supplementary Materials.

## Supplementary Materials

The following are available online at https://www.mdpi.com/article/10.3390/genes12050737/s1, Figure S1: Characterization of cell surface markers on iMSCs and UCB-MSCs, Figure S2: The top five KEGG pathways of upregulated and downregulated DEGs, Figure S3: Sashimi plots of the RNA-seq data for the three types of AS events (A5SS, A3SS, and MXE) that were differentially presented in iMSCs and UCB-MSCs, Table S1: Top 30 upregulated DEGs, Table S2: Top 30 downregulated DEGs, Table S3: Upregulated and downregulated DSGs with each AS type, Table S4: GO annotated DSGs with each AS type, analyzed by DAVID, Table S5: KEGG (A) and Reactome pathway analysis (B) of DSGs using GSEA, Table S6: Antibody list for FACS analysis, Table S7: PCR primer list for PCR validation, Table S8: GO annotated DEGs with each AS type, analyzed by DAVID.
