# Supplementary material for: Exploration of Alternative Splicing Events in Mesenchymal Stem Cells from Human Induced Pluripotent Stem Cells
Source: Genes (Basel). 2021 May 13;12(5):737. doi: 10.3390/genes12050737 (PMC8153164; doi:10.3390/genes12050737)

Supplementary Materials

Exploration of alternative splicing events in mesenchymal stem cells from human induced pluripotent stem cells

Ji Eun Jeong[1], Binna Seol[1], Han-Seop Kim[1], Jae Yun Kim[1,2], and Yee Sook Cho[1,2,*]

[1]Stem Cell Research Laboratory, Immunotherapy Research Center, Korea Research Institute of Bioscience and Biotechnology, 125 Gwahak-ro, Yuseong-gu, Daejeon, 34141, South Korea; [2]Department of Bioscience, KRIBB School, University of Science & Technology, 113 Gwahak-ro, Yuseong-gu, Daejeon 34113, South Korea

*Correspondence: Yee Sook Cho, Ph.D., Stem Cell Research Laboratory, Immunotherapy Research Center, Korea Research Institute of Bioscience and Biotechnology, 125 Gwahak-ro, Yuseong-gu, Daejeon 34141, South Korea, Tel: 82-42-860-4479, Fax: 82-42-860-4608, E-mail: june@kribb.re.kr

Supplemental Information

Supplemental information includes: Supplementary Figures S1-S3, Figure legends for figures S1-S3, Supplementary Tables S1-S8, and Table legends for Tables S1-S8.

Supplementary Figure S1. Characterization of cell surface markers on iMSCs and UCB-MSCs. Flow cytometric analysis of positive MSC markers (CD73, CD105, and CD90) and negative markers (CD34 and CD45) was performed in monolayer-cultured iMSCs and UCB-MSCs. The data are means ± SDs of three independent experiments.

Supplementary Figure S2. The top five KEGG pathways of upregulated and downregulated DEGs. KEGG pathway analysis was performed by using DAVID. The x-axis indicates the enrichment scores [−log10 (p value), p-value cut-off of 0.05] for each KEGG pathway term. The y-axis shows detailed classification of the KEGG pathway terms.

Supplementary Figure S3. Sashimi plots of the RNA-seq data for the three types of AS events (A5SS, A3SS, and MXE) that were differentially presented in iMSCs and UCB-MSCs. The sashimi plot was constructed with the MISO package. The vertical axis on the left side of the main panel shows the counts of RNA-Seq reads spanning the junctions in each region. The genomic coordinates for individual splice sites are shown at the top, and the schematic of each splicing event is shown at the bottom. Red indicates iMSCs (C_MSC-1), and orange indicates UCB-MSCs (C_MSC-3). A5SS, alternative 5′ splice site; A3SS, alternative 3′ splice site; MXE, mutually exclusive exon.

Supplementary Table S1. Top 30 upregulated DEGs (iMSCs *vs* UCB-MSCs).

Supplementary Table S2. Top 30 downregulated DEGs (iMSCs *vs* UCB-MSCs).

Supplementary Table S3. Upregulated and downregulated DSGs with each AS type (iMSCs

vs UCB-MSCs). A5SS, alternative 5′ splice site; A3SS, alternative 3′ splice site; MXE, mutually exclusive exon; RI, retained intron.

Supplementary Table S4. GO annotated DSGs with each AS type, analyzed by DAVID. CC, cellular component; BP, biological process; MF, molecular function.

Supplementary Table S5. KEGG (A) and Reactome pathway analysis (B) of DSGs using GSEA.

Supplementary Table S6. Antibody list for FACS analysis.

Supplementary Table S7. PCR primer list for PCR validation.

Supplementary Table S8. GO annotated DEGs with each AS type, analyzed by DAVID. CC, cellular component; BP, biological process; MF, molecular function.

Supplementary Table S1. Top 30 up-regulated DEGs (iMSCs *vs* UCB-MSCs).

Genes	Description	Fold change	Normalized RC (log2)	
		iMSCs/UCB-MSCs	iMSCs	UCB-MSCs
PAX8	Paired box 8	1162.788439	2431.126	2.090772713
LOC102724034	Uncharacterized LOC102724034	909.7359569	11493.04	12.63338224
ADAMTS7P1	ADAMTS7 pseudogene 1	476.4683437	91912.08	192.9027987
UBE2Q2P2	Ubiquitin conjugating enzyme E2 Q2 pseudogene 2	309.2364762	19229.02	62.1822453
L1CAM	L1 cell adhesion molecule	300.8134985	946.0126	3.144847651
KRT19	Keratin 19	274.8743749	9847.881	35.82684136
NDN	Necdin, MAGE family member	264.6554755	553.4132	2.091070253
FOXL2	Forkhead box L2	260.6615362	545.159	2.091444068
CLDN1	Claudin 1	241.4819778	1523.421	6.308631114
GOLGA6L10	Golgin A6 family-like 10	200.3113348	37796.23	188.6874069
SYNDIG1	Synapse differentiation inducing 1	195.4200298	408.736	2.091576958
SPP1	Secreted phosphoprotein 1	194.4936593	611.9382	3.146314233
DSC3	Desmocollin 3	179.4317136	2645.516	14.7438575
GOLGA6L17P	Golgin A6 family-like 17, pseudogene	176.7234659	15460.51	87.48417121
PDGFB	Platelet derived growth factor subunit B	167.5398937	350.4458	2.091715453
IGFN1	Immunoglobulin-like and fibronectin type III domain containing 1	150.5769441	632.6439	4.201465815
CPXM1	Carboxypeptidase X (M14 family), member 1	137.094866	286.7377	2.091527863
GALNT14	Polypeptide N-acetylgalactosaminyltransferase 14	133.9763653	421.6135	3.146924196
CNIH3	Cornichon family AMPA receptor auxiliary protein 3	132.5495956	417.0128	3.146088858
PPP1R14A	Protein phosphatase 1 regulatory inhibitor subunit 14A	127.855137	1480.783	11.58172632
TBX1	T-box 1	127.0366961	533.5866	4.200255369
FHOD3	Formin homology 2 domain containing 3	126.8895917	1469.575	11.58152673
GOLGA6L9	Golgin A6 family-like 9	117.3798539	35017.43	298.3257075
CELSR1	Cadherin EGF LAG seven-pass G-type receptor 1	115.8810893	975.6179	8.419129724
CDCP1	CUB domain containing protein 1	115.5766919	6455.961	55.85867464
RRAD	Ras-related associated with diabetes	103.9473972	327.108	3.146860571
NCAM2	Neural cell adhesion molecule 2	102.0849736	1397.634	13.69089181
OXTR	Oxytocin receptor	96.22165781	1824.4	18.96039185
LYPD1	LY6/PLAUR domain containing 1	95.58487721	5137.762	53.75077969
LOC727751	Golgin A2 pseudogene	89.91300922	46728.6	519.7090372

Supplementary Table S2. To 30 down-regulated DEGs (iMSCs *vs* UCB-MSCs).

Genes	Description	Fold change	Normalized RC (log2)	
		iMSCs/UCB-MSCs	iMSCs	UCB-MSCs
POSTN	Periostin	0.000471	88.38359	187753.3
MEG3	Maternally expressed 3 (non-protein coding)	0.000626	1.761836	2816.614
SULF1	Sulfatase 1	0.000884	12.05229	13629.58
ADAMTS2	ADAM metallopeptidase with thrombospondin type 1 motif 2	0.000942	7.763983	8240.488
FAP	Fibroblast activation protein alpha	0.000951	6.049402	6360.943
BGN	Biglycan	0.001135	25.77467	22703.18
OLFM2	Olfactomedin 2	0.00127	7.76366	6112.082
COL1A2	Collagen type I alpha 2	0.001353	373.979	276400.2
GALNT5	Polypeptide N-acetylgalactosaminyltransferase 5	0.001415	7.763323	5487.979
COL5A3	Collagen type V alpha 3	0.00142	15.48205	10906.59
ELN	Elastin	0.002099	5.18955	2472.944
SIX2	SIX homeobox 2	0.00233	3.474457	1491.481
NUPR1	Nuclear protein 1, transcriptional regulator	0.002564	10.33443	4030.008
LMO2	LIM domain only 2	0.002656	2.617027	985.4647
NPR3	Natriuretic peptide receptor 3	0.002788	9.476547	3398.54
LINC01133	Long intergenic non-protein coding RNA 1133	0.003018	5.188487	1719.19
HEY2	Hes related family bhlh transcription factor with YRPW motif 2	0.003128	1.760299	562.7336
MGP	Matrix Gla protein	0.003221	5.188425	1610.62
ZNF423	Zinc finger protein 423	0.003645	3.473362	952.7971
COL3A1	Collagen type III alpha 1	0.004034	703.3112	174337.5
ITGBL1	Integrin subunit beta like 1	0.004251	40.34947	9490.773
CXCL12	C-X-C motif chemokine ligand 12	0.004399	13.76451	3128.754
ITGA8	Integrin subunit alpha 8	0.004591	2.61821	570.2804
CRLF1	Cytokine receptor like factor 1	0.005155	1.759524	341.3509
ABCA6	ATP binding cassette subfamily A member 6	0.005405	1.759448	325.5381
IGFBP1	Insulin like growth factor binding protein 1	0.005732	18.90693	3298.385
ABCA8	ATP binding cassette subfamily A member 8	0.005773	8.615749	1492.537
CD248	CD248 molecule	0.005824	19.76897	3394.479
RRAGB	Ras related GTP binding B	0.006351	1.760303	277.1727
FLJ32255	Uncharacterized LOC643977	0.006475	1.76078	271.9357

Supplementary Table S3. Upregulated and downregulated DSGs with each AS type (iMSCs *vs* UCB-MSCs). A5SS, alternative 5′ splice site; A3SS, alternative 3′ splice site; MXE, mutually exclusive exon; RI, retained intron.

AS types	Upregulated genes	Downregulated genes
SE	*ACTB, BCAR1, BCS1L, BCS1L, BCS1L, CHN1, CNN2, CNN2, DBN1, EXOSC3, FAM171A1, FAT1, INO80E, LINC00623, LINC00623, LRR1, MGLL, MYL6, NASP, NPIPB3, NPIPB5, OR2A1-AS1, OR2A1-AS1, PALM, PFKFB3, PIN4, PIN4, PLEKHO1, PPAPDC1A, S100A13, SLC29A1, STYXL1, TAGLN, TIPIN, TRPT1, TRPT1, TRPT1, TUBA1A, TUBA1B, WNT5B, YIF1B, ZNHIT1*	*ABCC1, AFF3, BEST1, CALU, CALU, CALU, CCNI, CD164, CD164, CD164, CD46, EXTL2, EXTL2, FHL1, GSTM1, KIAA1462, LAMP2, LINC01123, LRRC32, MYCT1, NAB1, NAB1, NRP1, PXDN, SLC4A7, SUSD5, TFPI, TFPI, USP33*
A5SS	*C7orf50, GUK1, INO80E, ITPA, MRPL52, NDUFS5, RPL23AP7, TAGLN, TRPT1, TUBA1A, YIF1B, YIF1B, ZNHIT1*	*CCDC80, COL5A1, CTSL, CTSL, CTSL, CTSL, CTSL, DAG1, FKBP10, GREM1, MYNN, SPIN2B*
A3SS	*ADAM19, AP1G2, EMC9, EMC9, FBLIM1, PDLIM7, PRNP, S100A13, S100A13, SNRPB, TRPT1*	*CD164, COL12A1, COL5A1, COL6A2, LARP4, LTBP1, NR3C1, RGS4, TOR1AIP1, ZFP91*
MXE	*GUK1, RPS3, TCF3, TPM2, TUBA1A*	*APLP2, CALU, COL5A1, GNPDA2, IRAK1, IRAK1, ITGB5, PLAGL1, WASF3, ZBTB38*
RI	*AEN, AP1G2, AP1G2, AP1G2, ATPIF1, BEND7, CMTM4, GAS2L1, GUK1, GUK1, HCFC1R1, INO80E, MOK, MRPL52, S100A13, SPHK1*	*CCDC80, CD164, CEP350, GREM1, GREM1, MXRA8, NPTX1, P3H1, P3H1, P3H1, PDLIM5, PKD2, SEPT10, TGFBI, TGFBI, TNS3*

Supplementary Table S4. GO annotated DSGs with each AS type analyzed by DAVID. CC, cellular component; BP, biological process; MF, molecular function.

GO category		AS types				
		**SE**	**A5SS**	**A3SS**	**MXE**	**RI**
**CC**	Nucleolus	*ABHD14B, ACIN1, BCAR1, CTSB, DAXX, DEDD2, ENC1, EXOSC3, HJURP, HNRNPR, HSPA8, INO80E, MDM2, MORF4L2, PIN4, PRMT2, RBM4, RBM4B, RPAIN, RPS3A, RREB1, STK35, TFIP11, TRA2A, ZNF146, ZPR1*	*ADARB1, INO80E, NSUN5P1, RPS14, RPS2, SNAPC5, SNRPB2*	*CDV3, CIRBP, ILF3, MKNK2, NOL8, PHTF1, RFC1, RPS2, SRP19, SRSF5, TRA2A, ZCCHC11*	*CTSB, EEF1D, EEF1D, MDM2, RPS2, RPS3*	*ACADVL, ADARB1, ADARB1, AEN, BRWD1, DDX17, DDX24, DDX5, GNL3, HLTF, INO80E, MED1, NOL12, NSUN5P2, PHF6, RBM3, RPL10A, RPS2, SRSF5, TRA2A, WDR46*
	Focal adhesion	*ACTB, ACTN1, BCAR1, CD151, CD46, CNN2, DNM2, FAT1, FHL1, HNRNPK, HSPA8, NRP1, PCBP2, PLEC, RPS3A, RSU1, USP33*	*CD151, CTTN, DAG1, FLII, RPL6, RPS14, RPS16, RPS2*	*CD44, FBLIM1, GIT2, PDLIM7, RPS2*	*ACTN1, DCAF6, ITGB5, P4HB, RPLP0, RPS2, RPS3*	*RND3, RPL10A, RPS2, TNS3*
**BP**	SRP-dependent cotranslational protein targeting to membrane	*RPS24, RPS3A, SEC61A1*	*RPL6, RPS14, RPS16, RPS2*	*RPS2, RPS2, SRP19*	*RPLP0, RPS2, RPS2, RPS20, RPS3, SRP9*	*RPL10A, RPL10A, RPL13, RPS2*
	RNA splicing	*ACIN1, HNRNPC, MTERF4, RBM4, RBM4B, SNRNP70, SON, SRSF4, TFIP11, ZNF638, ZPR1*	*HNRNPH3, TARDBP*	*DDX39B, PTBP3, QKI, SNRPB*	*HNRNPC,*	*DDX39B, PPP1R8, SON*
**MF**	poly(A) RNA binding	*ACIN1, ALG13, ATP5C1, CAST, DHX30, GIGYF2, HNRNPAB, HNRNPC, HNRNPH1, HNRNPK, HNRNPR, HSP90AA1, HSPA8, LTA4H, MRPL32, NDUFV3, PCBP2, PIN4, PLEC, R3HDM2, R3HDM2, RBM4, RBM4B, RPS24, RPS3A, SLC3A2, SNRNP70, SON, SRSF4, TRA2A, YTHDF1, YTHDF3, ZFP36L1, ZNF638*	*ADARB1, C7orf50, DSP, HNRNPH3, RPL6, RPS14, RPS16, RPS2, TARDBP, WDR6*	*CDK13, CIRBP, DDX39B, ILF3, LARP4, NOL8, PTBP3, QKI, R3HDM2, RBM26, RPS2, SERBP1, SNRPB, SRP19, SRSF5, TRA2A, ZCCHC11*	*CLNS1A, CSDE1, EIF4A1, HNRNPC, MTHFSD, P4HB, R3HDM2, RBMX, RPLP0, RPS2, RPS20, RPS3*	*ADARB1, DDX17, DDX24, DDX39B, DDX5, GNL3, HLTF, HNRNPH1, KMT2C, NAP1L1, NOL12, PHF6, RBM3, RPL10A, RPL13, RPS2, SON, SRRM2, SRSF5, TRA2A, WDR46*
	RNA binding	*EXOSC3, FARSB, HNRNPAB, HNRNPC, HNRNPH1, HNRNPK, HNRNPR, PCBP2, RBM4, RPS3A, SNRNP70, SON, YTHDF3, ZNF638*	*ADARB1, HNRNPH3, NSUN5P1, RPL6, RPS14, RPS16, RPS2, TARDBP*	*CIRBP, ILF3, KIAA0907, NR3C1, PRKCSH, PTBP3, QKI, RBM26, RPS2, SNRPB, SRSF5*	*HNRNPC, PSMA6, RBMX, RPS2, RPS20, RPS3, SRP9*	*ADARB1, DDX17, HNRNPH1, NSUN5P2, PPP1R8, RBM3, RPL10A, RPL13, RPS2, SON, SRSF5,*

Supplementary Table S5. KEGG (A) and Reactome pathway analysis (B) of DSGs using GSEA.

A. KEGG pathway

**Gene Set Name**	**p-value**	**FDR q-value**
Spliceosome	1.01 e^{-9}	1.89 e^{-7}
Ribosome	2.95 e^{-8}	2.75 e^{-6}
Lysosome	4.17 e^{-5}	1.94 e^{-3}
Focal adhesion	5.45 e^{-5}	2.03 e^{-3}
Tight junction	8.58 e^{-5}	2.66 e^{-3}
Protein export	1.04 e^{-4}	2.77 e^{-3}
Endocytosis	1.39 e^{-4}	3 e^{-3}
Arrhythmogenic right ventricular cardiomyopathy	1.45 e^{-4}	3 e^{-3}
ECM receptor interaction	2.51 e^{-4}	4.68 e^{-3}

B. Reactome pathway

**Gene Set Name**	**P-value**	**FDR q-value**
Metabolism of RNA	5.82E-17	8.92E-14
Nervous system development	2.77E-13	2.12E-10
Post-translational protein modification	5.05E-13	2.58E-10
Translation	3.48E-12	1.33E-09
SRP-dependent cotranslational protein targeting to membrane	4.26E-11	1.3E-08
Extracellular matrix organization	2.2E-10	5.61E-08
Developmental biology	5.69E-10	1.25E-07
Eukaryotic translation elongation	5.97E-09	1.14E-06
Processing of capped intron containing premRNA	7.32E-09	1.25E-06

Supplementary Table S6. Antibody list for FACS analysis.

Protein	Company (Cat. No.)	Dilution
CD73-PE	Miltenyl Biotec (130-095-182)	1 : 11
CD105-PE	Miltenyl Biotec (130-112-321)	1 : 11
CD90-PE	Miltenyl Biotec (130-114-860)	1 : 11
CD34/CD45 Cocktail, FITC, PE	Thermo Fisher (MA1-12221)	1 : 11
FITC Mouse IgG2a, Isotype Ctrl	BioLegend (400210)	1 : 200
PE Mouse IgG1 isotype control	Miltenyl Biotec (130-113-200)	1 : 50

Supplementary Table S7. PCR primer list for PCR validation.

Gene name		5'-3' sequence	length
TRPT1 **SE**	LEFT PRIMER	CATGCGGTCCCATTGTGAAA	127bp/238bp
	RIGHT PRIMER	TTCTCTGGAGCTGTGCTTGG	
CNN2**_SE**	LEFT PRIMER	CCTGTCCAACTTCATCAAGGC	222bp/338bp
	RIGHT PRIMER	CCATCTGGAGGCTGATGGTC	
AP1G2**_RI**	LEFT PRIMER	GACTGCAGTGCACATGATCC	313bp/142bp
	RIGHT PRIMER	AGGGCTTCGTTCGCAGAG	
**GAPDH**	LEFT PRIMER	GAAGGTGAAGGTCGGAGTC	226bp
	RIGHT PRIMER	GAAGATGGTGATGGGATTTC	

Supplementary Table S8. GO annotated DEGs with each AS type analyzed by DAVID. CC, cellular component; BP, biological process; MF, molecular function

**GO category**		**AS types**				
		**SE**	**A5SS**	**A3SS**	**MXE**	**RI**
**CC**	Focal adhesion	*ACTB, BCAR1, CD46, CNN2, FAT1, FHL1, NRP1, USP33*	*DAG1*	*FBLIM1, PDLIM7*	*ITGB5, RPS3*	*TNS3*
	Extracellular exosome	*ACTB, CNN2, EXTL2, FAM171A1, FAT1, LAMP2, PIN4, TUBA1B*	*CTSL*	*COL12A1, PRNP, SNRPB*	*ITGB5*	*BEND7, PKD2, TGFBI*
**BP**	Extracellular matrix organization	*PXDN*	*CCDC80, COL5A1, DAG1*	*COL5A1, COL6A2*	*COL5A1, ITGB5*	*CCDC80, TGFBI*
	Cell adhesion	*BCAR1, CD164, FAT1, KIAA1462, SUSD5*	*COL5A1*	*CD164, COL12A1, COL5A1, COL6A2*	*COL5A1, ITGB5*	*CD164, TGFBI*
**MF**	Actin binding	*CNN2, DBN1, TAGLN*	*DAG1, TAGLN*	-	*TPM2, WASF3*	*PDLIM5*

Supplementary Figure S1. Characterization of cell surface markers on iMSCs and UCB-MSCs. Flow cytometric analysis of positive MSC markers (CD73, CD105, and CD90) and negative markers (CD34 and CD45) was performed in monolayer-cultured iMSCs and UCB-MSCs. The data are means ± SDs of three independent experiments.

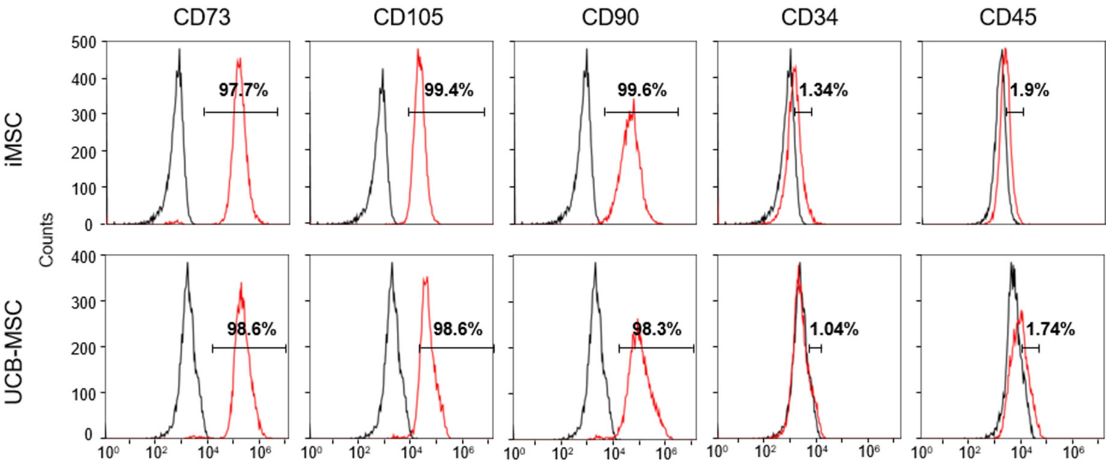

Supplementary Figure S2. The top five KEGG pathways of upregulated and downregulated DEGs. KEGG pathway analysis was performed by using DAVID. The x-axis indicates the enrichment scores [−log10 (p value), p-value cut-off of 0.05] for each KEGG pathway term. The y-axis shows detailed classification of the KEGG pathway terms.

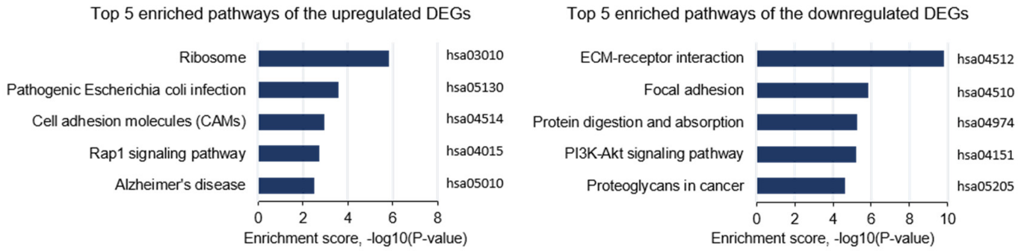

Supplementary Figure S3. Sashimi plots of the RNA-seq data for the three types of AS events (A5SS, A3SS, and MXE) that were differentially presented in iMSCs and UCB-MSCs. The sashimi plot was constructed with the MISO package. The vertical axis on the left side of the main panel shows the counts of RNA-Seq reads spanning the junctions in each region. The genomic coordinates for individual splice sites are shown at the top, and the schematic of each splicing event is shown at the bottom. Red indicates iMSCs (C_MSC-1), and orange indicates UCB-MSCs (C_MSC-3). A5SS, alternative 5′ splice site; A3SS, alternative 3′ splice site; MXE, mutually exclusive exon.

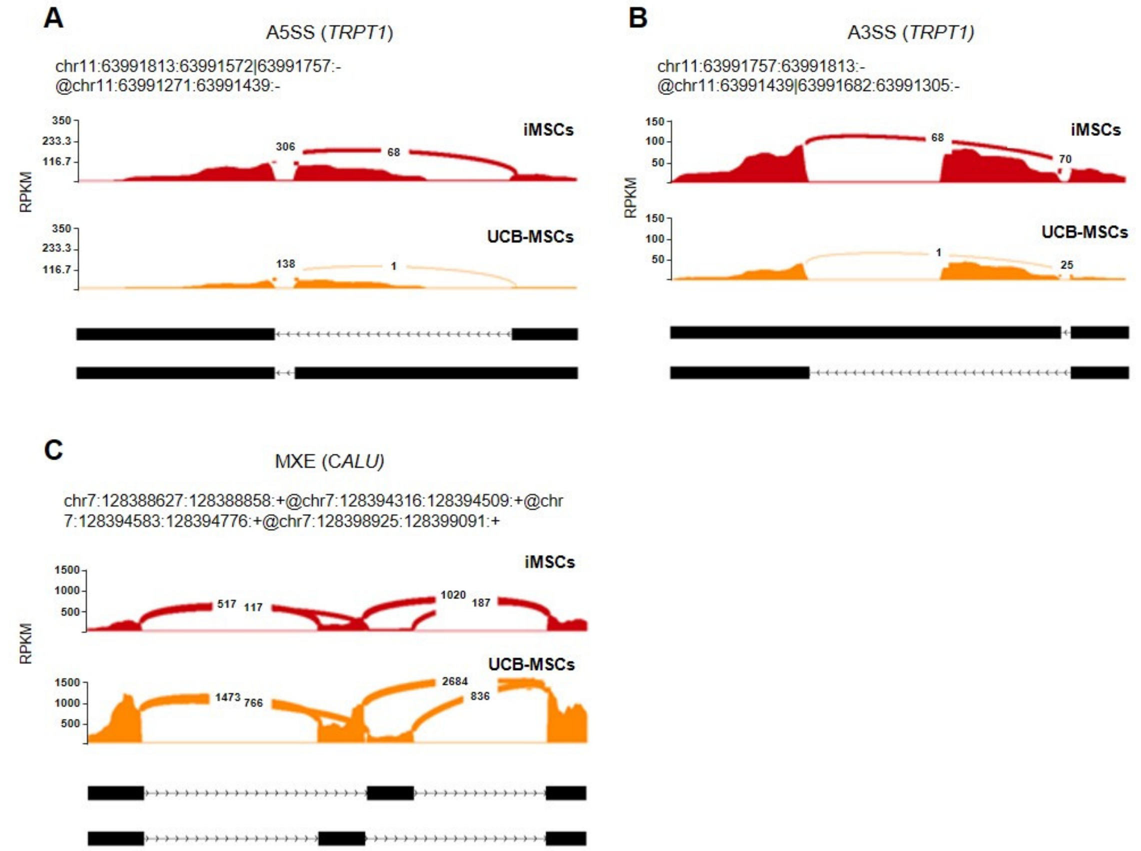

Supplement: Supplementary file 1 [file genes-12-00737-s001.zip › genes-1209998-supplementary.pdf]
